# Methionine Partially Replaced by Methionyl-Methionine Dipeptide Improves Reproductive Performance over Methionine Alone in Methionine-Deficient Mice

**DOI:** 10.3390/nu10091190

**Published:** 2018-09-01

**Authors:** Qiong Chen, Wenting Dai, Yalu Sun, Fengqi Zhao, Jianxin Liu, Hongyun Liu

**Affiliations:** 1 College of Animal Sciences, Zhejiang University, Hangzhou 310058, China; 11717002@zju.edu.cn (Q.C.); daiwenting_2012@sina.com (W.D); 21717059@zju.edu.cn (Y.S.); fzhao@uvm.edu (F.Z.); liujx@zju.edu.cn (J.L.); 2 Department of Animal and Veterinary Sciences, University of Vermont, Burlington, VT 05405, USA

**Keywords:** methionyl-methionine, methionine, amino acid, reproduction, AKT/mTOR signaling pathway

## Abstract

Decreased protein breakdown in pregnant women results in lower concentration of methionine (Met) in plasma, causing pregnancy-related metabolic disturbance. Its dipeptide methionyl-methionine (Met-Met) may exert positive influence in fetal development. This study mainly investigated whether Met-Met can be used as part of free Met to promote reproductive outcomes in mice and the underlying mechanisms. Met-deficient pregnant mice were treated with Met alone or with Met-Met during pregnancy. Daily intraperitoneal injection of 35% dietary Met in pregnant mice was the best dose among the 15–45% doses. Embryo development and newborn birth weight were enhanced when 25% of the Met in the 35% Met group was replaced with Met-Met. Met-Met replacement had higher plasma insulin, glucose, and free amino acids (AA) concentrations. Besides, in the placenta, the AA transporter mRNA abundances and peptide transporters (PhT1 and PepT1) protein levels were higher in Met-Met treatment group. Moreover, Met-Met increased 4E-BP1, S6K1 and AKT/mTOR phosphorylation. These results suggest that Met-Met could be used as a partial source of Met to promote reproductive outcomes in Met-restricted pregnant mice, which might be mediated by promoting nutrient availability and activating AKT/mTOR-mediated signaling pathway.

## 1. Introduction

Methionine (Met) is an essential amino acid (EAA) required for protein synthesis and is also a key source of methyl groups for methylation [1], such as the methylation of DNA, epigenetics, biogenic amines and phospholipids during development [2]. Studies in humans have suggested that perturbations in maternal Met metabolism may lead to pregnancy-related disorders, including spontaneous abortion, placental abruption, and premature delivery [3,4]. The rate of protein breakdown is decreased during normal human pregnancy, leading to lower plasma concentrations of all AAs, including Met [4,5]. Recent data have indicated that the concentration of total homocysteine, an intermediate of Met metabolism, is lower in maternal than in non-pregnant women during the 3rd trimester in plasma [6]. The low Met concentration may have a negative effect on embryo development.

Short peptides have drawn increasing attention as new sources of nutrients. The uptake of short peptides by oligo/dipeptide transporters in cells provides a more effective and energy-saving intracellular source of AAs than the uptake of free AAs (FAAs) [7,8]. It was first found in 1996 that small peptide-containing Met could serve as a source of free Met for milk protein synthesis, and the efficiency of some Met dipeptides was much higher than the equivalent FAA in mouse mammary gland tissue explants [9]. Recently, we identified that small peptides containing Met, Lys, or Phe could promote αS1-casein and β-casein synthesis in bovine mammary gland explants [10,11,12,13]. Apart from its role as protein synthesis substrates, small peptides may also function as signaling molecules to promote cell cycle transition and AA absorption [12,13]. Although the role of dipeptides in milk protein synthesis *in vitro* is clearly established, little is known about its role in fetal development. We hypothesize that partially supplementing pregnant dams with the Met dipeptide methionyl-methionine (Met-Met) is more effective than supplementing them with only free Met in promoting embryo development and improving reproductive outcomes. In this study, pregnant mice with dietary Met restriction were supplemented with Met alone or with Met-Met by intraperitoneal (i.p.) injection to investigate the effect and mechanism of Met-Met on reproductive outcomes in mice.

## 2. Materials and Methods

### 2.1. Peptide Stability in Biological Fluids

Biological fluids, including simulated gastric fluid (SGF) and intestinal fluid (SIF), were prepared and Met-Met stability in biological fluids was measured according to previously described procedures [14].

### 2.2. Animals

The use of animals in this study was approved by the Institutional Animal Care and Use Committee of Zhejiang University. ICR and Nu/nu mice were purchased from the Shanghai Laboratory Animal Center. Female virgin mice of approximately 30 g and 9 weeks of age were mated, and the presence of seminal plugs indicated a successful mating. The day that seminal plugs were found was designated embryonic day (E) 0. The mice were housed individually in a controlled environment at 21 ± 3 °C under specific pathogen-free conditions with a 12h light-dark cycle and ad libitum access to food and water.

### 2.3. In Vivo Optical Imaging

Nine Nu/nu mice aged 6 weeks were used to observe the disappearance of fluorescein isothiocyanate-labeled Met-Met (FITC-Met-Met) in the body, and were randomly divided into the following groups (each group *n* = 3): blank control, FITC-Met-Met, and Free-FITC, respectively. A total of 100 µL of saline, 23.05 µg/mL FITC-Met-Met (GL Biochem, Shanghai, China), or 15 µg/mL Free-FITC (GL Biochem) was injected into the peritoneal cavity of nu/nu mice in three groups, respectively. At different time intervals (0.5, 2, 5, and 24 h), mice were anesthetized with halothane and placed in a fluorescence imaging system (MAESTRO, CRI, USA) in which the “blue” filter was set for excitation and the tunable filter was automatically increased from 465 to 520 nm in 10 nm stepwise increments. The Maestro software (CRi) was used to unmix the fluorescence signals. Light intensity was indicated by pseudocoloring (red for the highest intensity and blue for the lowest intensity), superimposed over the grayscale body surface.

### 2.4. Maternal Studies

Experiment I: Forty pregnant ICR mice were randomly assigned to 5 groups (*n* = 8). The mice were fed a Met-supplemented or Met-deficient diet (Harlan, Table S1). The diets were modified standard AA-defined diets containing the amounts of choline, vitamins and minerals recommended for the AIN-93G formulation [15]. The AA contents of the diets were similar to that detailed in Rogers and Harperin [16], except that Met was absent in the treatments groups’ diets. From E 0, the control group (Control) was fed a Met-supplemented diet and received i.p. injections of 0.4 mL of saline, whereas the other five groups were fed the same diet without Met and received daily i.p. injections of 15, 25, 35 or 45% of the Met content contained in the control diet (39.2 mg based on 4.8 g/day dry matter intake) in 0.4 mL of saline at 10:00 a.m. (Figure S1A). Because most of the fetuses of mice fed with the Met-deficient diet that received i.p. injections of 0, 5, 55, and 65% of the Met were aborted during pregnancy, they were not included in this study. The food intake (g/24 h) levels and body weights of the dams were recorded at different stages of gestation. At E 17, all mice were euthanized via CO_2_ asphyxiation at the same time of day (15:00 to 16:00) to avoid circadian variations in gene expression. Livers were immediately collected, snap frozen in liquid nitrogen, and stored at −80 °C until subsequent analysis for Met metabolites [17]. Embryos were weighed and litter numbers were counted. The animals that aborted before E 17 were excluded from the experiment.

Experiment II: Fifty-six pregnant ICR mice were randomly assigned to seven groups (*n* = 8). Groups 1 (Control) and 2 (35% Met) were treated as described for the control group and 35% Met group in experiment I, respectively. The other five groups (5, 15, 25, 35, and 45% Met-Met) were treated as described for the 35% Met group, except that the 5, 15, 25, 35, or 45% of free Met in the injected solution was replaced with Met-Met (i.e., 95–55% of the total Met provided as free Met + 5–45% of the total Met provided as Met-Met; Figure S1B). The observations of embryonic development were carried out as described for experiment I.

Experiment III: Forty-eight pregnant ICR mice were randomly divided into three groups (*n* = 16) and received treatments as described for the control, 35% Met, and 25% Met-Met groups in experiment II (Figure S1C). At E 17, half of the animals in each group (Eight mice) were euthanized via CO_2_ asphyxiation, and then maternal blood was collected into EDTA tubes at the posterior orbital venous plexus and centrifuged at 2000 g for 10 min at 4 °C to acquire plasma. The plasma was snap frozen in liquid nitrogen and stored at −80 °C until subsequent analysis for insulin (Insulin Mouse ELISA kit), leptin (Leptin Mouse ELISA kit) and glucose (Glucose Colorimetric Detection Kit) using kits from Nanjing Jiancheng Bioengineering Institute (Nanjing, China). The placentas in each horn next to the cervix unit were rapidly removed from these animals, and fetuses were carefully collected and sampled for blood. The remaining 8 dams in the two treatment groups were switched to the control diet without i.p. injections to avoid any abortion. Litters were weighed soon after parturition and before milk consumption.

### 2.5. Amino Acid Analysis

FAAs were extracted from 350 µL of plasma samples with 350 mL of 10% sulfosalicylic acid by vigorous shaking for 1 h [18]. The suspension was then centrifuged at 10,000 g for 10 min at 4 °C, filtered with 0.22µm syringe filters, and analyzed with a Hitachi L-8900 amino acid analyzer (Hitachi, Tokyo, Japan).

### 2.6. Quantitative Reverse Transcription-PCR (qRT-PCR) and Western Blot Analysis

The qRT-PCR procedures [12] and protein level measurements by Western blot analysis [19] were carried out according to previously described methods. The primer sequences and sources of the antibodies are listed in Table S2. The mRNA abundance and protein level of individual genes were normalized against the levels of the internal control gene β-actin and the phosphorylation level was calculated as the ratio of the phosphorylated to total protein level.

### 2.7. Statistics

The results are presented as the mean ± SEM. Comparisons among groups were analyzed by one-way or two-way ANOVA using SPSS software (version 20; IBM, Armonk, NY, USA). Differences between groups were analyzed post hoc using the LSD comparison. Animal litter was used as an observation unit for all offspring analyses. Differences of *p* < 0.05 were considered significant.

## 3. Results

### 3.1. Stability of Met-Met in Biological Fluids and In Vivo Biodistribution

**Figure 1:**
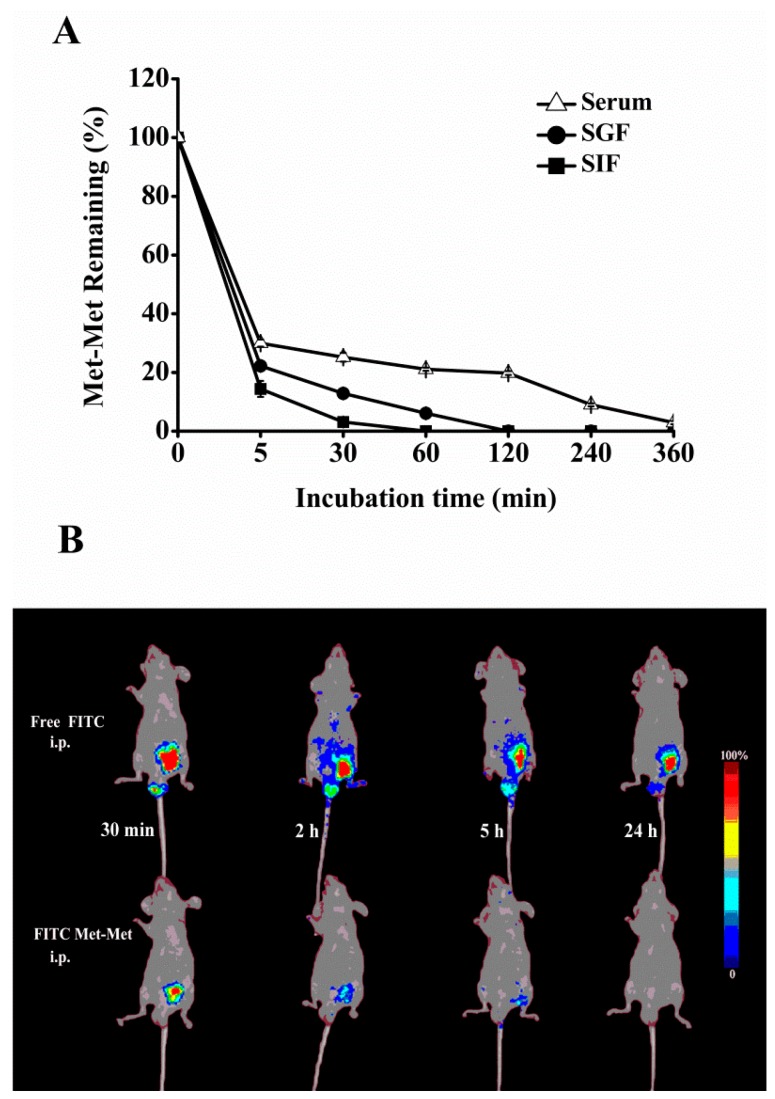
Stability of methionyl-methionine (Met-Met) in biological fluids and *in vivo* spectral fluorescence imaging of nu/nu mice. Met-Met disappearance (%) was measured after Met-Met was incubated in gastric fluid (SGF), intestinal fluid (SIF), or serum for 5–360 min in vitro (**A**). Live animal fluorescence imaging was performed in nu/nu mice after they were intraperitoneally (i.p.) injected with either Free-FITC or FITC-labeled Met-Met for 30 min–24 h (**B**). Data with error bars represent the mean ± SEM, *n* = 3.

Met-Met was degraded quickly in SIF and SGF and became undetectable after 1–2 h of incubation (Figure 1A). However, Met-Met was more stable in serum than in SIF and SGF and retained 21% at 120 min of incubation. Furthermore, the absorption of Met-Met into the circulatory system after i.p. injection into the peritoneal cavity is shown in Figure 1B. FITC-Met-Met showed rapid absorption during the first 5 h and was not visible after 24 h; however, a considerable amount of Free-FITC was still accumulated in the abdomens of the Free-FITC-injected mice. Thus, we supplied Met-Met by daily i.p. injection in subsequent animal experiments.

### 3.2. Reproductive Performance with Different Levels of Free Met

**Figure 2:**
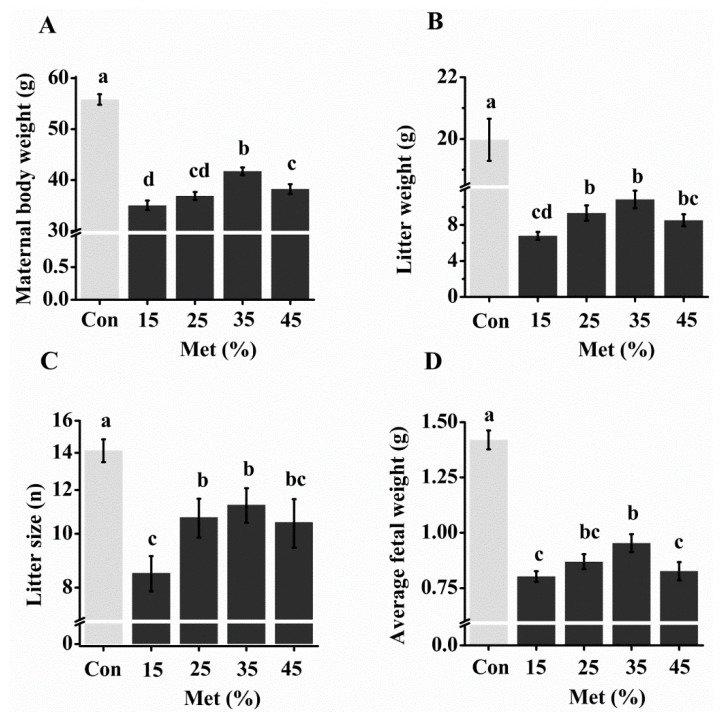
Maternal body weight (**A**), litter weight (**B**), litter size (**C**), and average fetal weight (**D**) at day 17 with different free methionine (Met) supplementation regimens. The control group (Con) was fed a diet with Met, whereas the 15–45% Met groups were fed a Met-deficient diet supplemented with 15, 25, 35, or 45% of the Met in the control diet by intraperitoneally injection from conception. The data are expressed as the mean ± SEM, *n* = 6–7. The different letters indicate significant differences, *p* < 0.05.

In experiment I, all dams that had consumed Met-deficient diets showed significantly lower body weights than those in the control group from day 8 of gestation, and the body weights of the Met-deficient dams were 66–77% of the control group weight at day 17 (Figure 2A; Table S3A). However, supplementation of 35% dietary Met by i.p. injection (35% Met group) showed the highest body weight of the 15–45% Met treatment groups, beginning at day 11 (Table S3A). Similarly, Met deficiency in diet significantly impaired embryo development, but the 25% and 35% Met groups showed the best embryo development among the Met treatment groups in terms of litter weight, litter size, and average fetal weight (Figure 2B–D). In addition, the Met treatments had no impact on the liver contents of Met metabolites compared with the control animals (Table S4). The food intake of the dams in the Met treatment groups decreased significantly (*p* < 0.001). Whereas the 35% Met group increased cumulative food intake at E 14 compared with the 15% Met group (*p* = 0.032), no difference existed among other Met groups (Table S3B,C). Taken together, we chose 35% of Met contained in the diet as a baseline in the following studies.

### 3.3. Reproductive Performance When Free Met Was Partially Substituted with Met-Met

**Figure 3:**
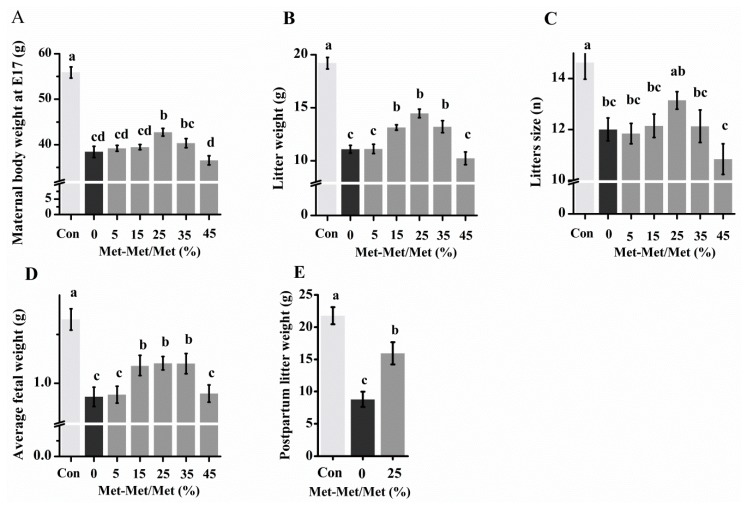
Maternal body weight (**A**), litter weight (**B**), litter size (**C**), and average fetal weight (**D**) at day 17 and postpartum litter weight (**E**) with different levels of methionine (Met) supplementation as methionyl-methionine (Met-Met). The control group (Con) was fed a diet with Met, and the 35% Met group was fed a Met-deficient diet supplemented with 35% of the Met in the control diet by intraperitoneallyinjection from conception. In the 5, 15, 25, 35, and 45% Met-Met groups, 5, 15, 25, 35, or 45% Met in the 35% Met group was replaced with Met-Met. Data are expressed as the mean ± SEM, *n* = 6–8. The different letters indicate significant differences, *p* < 0.05.

In experiment II, replacement of 5–45% of free Met in the 35% Met group with Met-Met did not reduce the body weight of the dams or fetal development (Figure 3; Table S5A). In fact, at E 17, the maternal body weight was significantly higher when 25% of Met were replaced with Met-Met (Figure 3A). Additionally, although there were still gaps in embryo development compared with the Met-sufficient control group, replacements of 15%, 25%, and 35% Met with Met-Met increased litter weight and average fetal weight compared with the 35% Met group (0% Met-Met group) (Figure 3B,D). No significant difference in litter size was seen between 25% Met-Met and the control group (Figure 3C). Furthermore, the 25% Met-Met group showed lower growth retardation at birth (*p* = 0.001, Figure 3E), and higher cumulative food intake at E 14 (*p* = 0.019, Table S5B,C) than the 35% Met group. Consequently, substitution of 25% Met into Met-Met was the top performer.

### 3.4. Amino Acids in the Plasma of Dams and the Associated Fetal with Different Sources of Met

**Figure 4:**
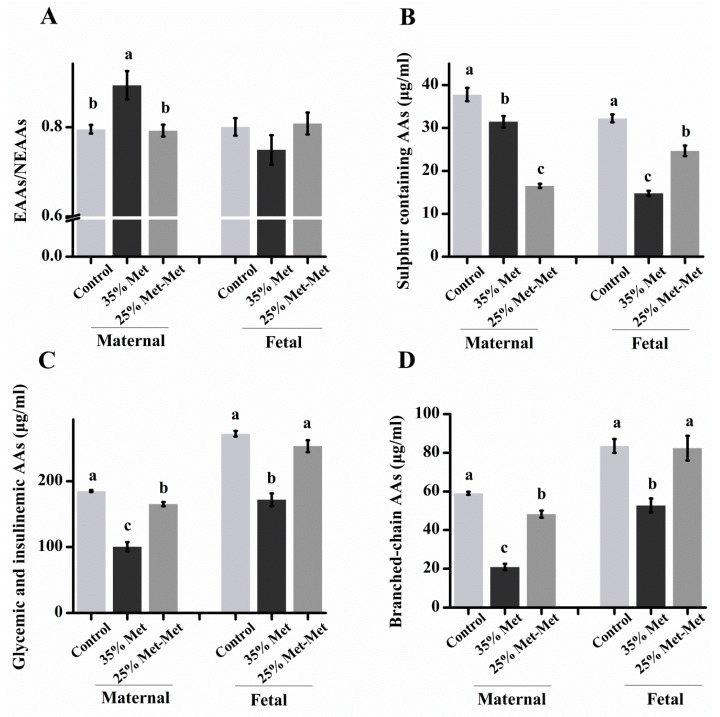
Concentrations of free amino acids (FAAs, μg/mL) in maternal and fetal plasma on day 17 of gestation in mice with different sources of methionine (Met) supplementation. The subfigures are maternal and embryo plasma EAAs/NEAAs (**A**), sulfur-containing AAs (**B**), glycemic and insulinemic AAs and (**C**), and branched-chain AAs (**D**). In the control group, dams were fed a diet supplemented with free Met. In the 35% Met group, dams were fed a Met-removed diet with intraperitoneally injection of 35% of the Met contained in the control diet. The 25% Met-Met group treated was as described for the 35% Met group, except that 25% of the injected Met was replaced with methionyl-methionine (Met-Met). Sulfur-containing AAs: Cys + Met, glycemic and insulinemic AAs: Val + Leu + Ile + Thr + Lys + Arg, and branched-chain AAs: Val + Leu + Ile. The data are expressed as the mean ± SEM, *n* = 8. The different letters indicate significant differences, *p* < 0.05.

In experiment III, at E 17, maternal dietary Met restriction induced a significant decrease in the concentrations of total FAAs and essential and nonessential AAs (EAAs and NEAAs) in the plasma of the dams, with 45% and 19% reductions in total FAAs in the 35% Met and 25% Met-Met groups, respectively (Table S6). The ratio of EAAs to NEAAs increased in the dams of the 35% Met group (*p* = 0.002), but no differences were found in the dams and fetuses of 25% Met-Met group compared with the control group (Figure 4A). Additionally, most of the EAAs and NEAAs, such as Ile, Lys, Val, Ala, Asp, Glu, Ser and Tyr, increased significantly in the maternal and fetal plasma in the 25% Met-Met group compared to those in the 35% Met group (Table S6). Moreover, the sum of the AAs reported to promote insulin secretion (i.e., Val + Leu + Ile + Lys + Thr + Arg) [20] was lower in the maternal and fetal plasma of the 35% Met group (*p* < 0.001) than that in the 25% Met-Met group (Figure 4C), so did the branched-chain AAs (Val + Leu + Ile; Figure 4D). Conversely, the concentrations of the sulfur-containing FAAs (i.e., Met + Cys) were significantly lower in the dams in the 25% Met-Met group (*p* < 0.001) than those in the 35% Met group (Figure 4B). However, the levels of sulfur-containing FAAs in fetal plasma were much higher in the 25% Met-Met group than those in the 35% Met group. Overall, the concentrations of all FAAs, especially Phe and Ser, were higher in the fetal plasma than those in the maternal plasma in all groups, including the control group (Table S6). The result indicated that supplementation with Met-Met significantly attenuated the FAAs imbalance both in the maternal and fetal blood circulation caused by Met deficiency.

### 3.5. Plasma Glucose and Insulin in Dams With Different Sources of Met

**Figure 5:**
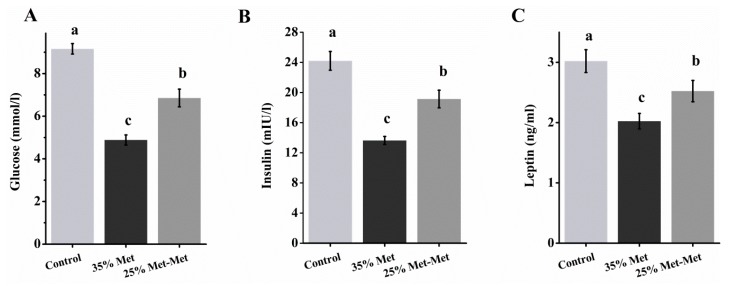
Maternal plasma concentrations of glucose (**A**), insulin (**B**), and leptin (**C**) at day 17 with different sources of methionine (Met) supplementation. In the control group, pregnant mice were fed a diet containing free Met. In the 35% Met group, pregnant mice were fed a Met-deficient diet with intraperitoneally injection of 35% of the Met contained in the control diet. The 25% Met-Met group was treated as described for the 35% Met group, except that 25% of the injected Met was replaced with methionyl-methionine (Met-Met). Data are expressed as the mean ± SEM, *n* = 8. The different letters indicate significant differences, *p* < 0.05.

Maternal Met-deficient diet intake decreased plasma glucose, insulin and leptin levels, but the 25% Met-Met group showed higher concentrations than the 35% Met group (*p* < 0.001, *p* = 0.02, and *p* < 0.001 for glucose, insulin, and leptin, respectively, Figure 5). The increased plasma glucose and insulin levels by Met-Met supplementation suggested a changed glucose metabolism in animals.

### 3.6. Nutrient Transporters in the Placenta with Different Sources of Met

#### 3.6.1. Amino Acid Transporters

**Figure 6:**
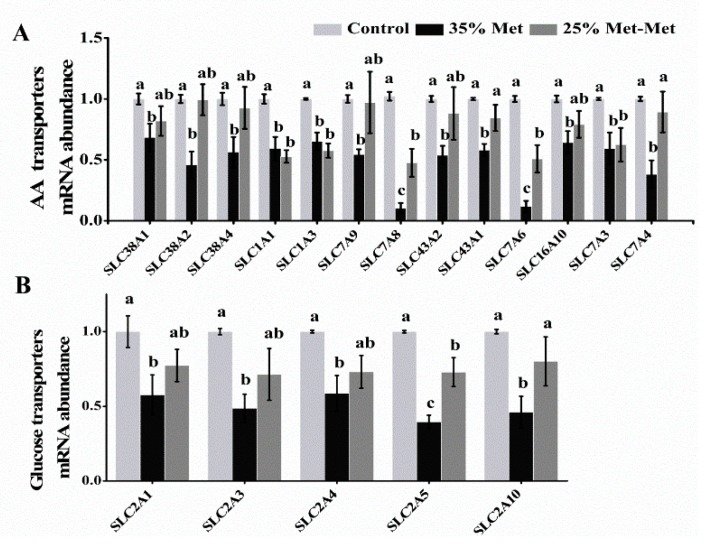
The AA transporters mRNA abundance (**A**) and glucose transporters (**B**) in the placenta at day 17 with different sources of methionine (Met) supplementation. The control group (Control) was fed a diet supplemented with Met, and the 35% Met group was fed a Met-restricted diet and received intraperitoneally injections with 35% of the Met in the control diet. The 25% Met-Met group was treated as the 35% Met group, with 25% of the Met replaced with methionyl-methionine (Met-Met). The data are expressed as the mean ± SEM; three placentas were selected randomly from each group, *n* = 3. The experiment was repeated three times. The different letters indicate significant differences, *p* < 0.05.

Compared to the control group, the 35% Met group had a markedly decreased mRNA abundance of AA transporters, including solute carrier (SLC) family members, SLC38A1, SLC38A2 and SLC38A4 in system A, SLC7A9 in system b^0,+^, SLC43A2 and SLC43A1 in system y^+^L, SLC16A10 in system T, and SLC7A4 in system y^+^ in the placenta at E 17 in experiment III (Figure 6A; Table S7). However, the 25% Met-Met group showed no difference. Additionally, the 25% Met-Met group showed an increased abundance of SLC7A8 in system L and SLC7A6 in system y^+^L compared with the 35% Met group. Furthermore, the mRNA abundance levels of SLC1A1 and SLC1A3 in system X^-^_AG_ and SLC7A3 in system y^+^ were decreased in both dietary Met-deficient groups compared with those in the control group. The results demonstrated that provision with Met-Met better promoted the abundance of amino acid transporters impaired by Met deficiency.

#### 3.6.2. Glucose Transporters

The mRNA abundance levels of facilitative glucose transporters SLC2A1, SLC2A3, SLC2A4, and SLC2A10 in the placenta at E 17 decreased significantly in the 35% Met group compared with those in the control group (Figure 6B; Table S7), whereas substitution of 25% of Met with Met-Met enhanced these abundance levels to the control. The mRNA abundance of SLC2A5 was lower in both treatment groups. The increased abundance of glucose transporters supplied with Met-Met in placenta indicated that higher glucose concentration may be transported into fetal blood.

#### 3.6.3. Peptide Transporters

**Figure 7:**
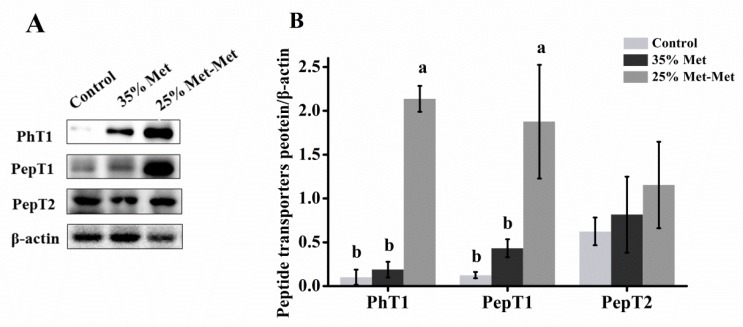
Representative immunoblots (**A**) and blot density analysis (**B**) of peptide/histidine transporter 1 (PhT1), peptide transporter 1 (PepT1), and peptide transporter 2 (PepT2) in the placenta of mice at day 17. In the control group, pregnant mice were fed a diet containing free Met. In the 35% Met group, pregnant mice were fed a Met-deficient diet with intraperitoneally injection of 35% of the Met contained in the control diet. The 25% Met-Met group was treated as described for the 35% Met group, except that 25% of the injected Met was replaced with methionyl-methionine (Met-Met). The data are expressed as the mean ± SEM; three placentas were selected randomly from each group, *n* = 3. The different letters indicate significant differences, *p* < 0.05.

The protein expression levels of peptide/histidine transporter 1 (PhT1) and peptide transporter 1 (PepT1) were significantly enhanced in the 25% Met-Met group compared with those in the control and 35% Met groups (Figure 7). The peptide transporter 2 (PepT2) was similar in three groups. The results indicated that part of Met-Met was absorbed as a whole by peptide transporters.

### 3.7. Signaling Molecules in the Placenta with Different Sources of Met

**Figure 8:**
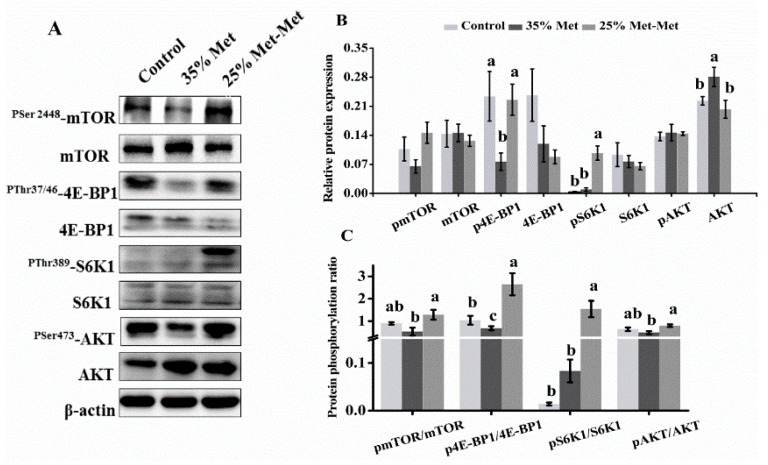
Expression of mTOR signaling proteins at day 17 in the placenta of mice supplemented with different sources of methionine (Met). The subfigures are representative immunoblots of the phosphorylated (P) forms and total (T) levels of the mTOR signaling proteins (**A**), the spot density analysis of the P forms and T levels of the mTOR signaling proteins (**B**), and the spot density ratio of the P:T forms of the mTOR signaling proteins (**C**). In the control group, pregnant mice were fed a diet containing free Met. In the 35% Met group, pregnant mice were fed a Met-deficient diet with intraperitoneally injection of 35% of the Met contained in the control diet. The 25% Met-Met group was treated as described for the 35% Met group, except that 25% of the injected Met was replaced with methionyl-methionine (Met-Met). The data are expressed as the mean ± SEM; three placentas were selected randomly from each group, *n* = 3. The different letters indicate significant differences, *p* < 0.05.

The protein expression levels of mTOR, pmTOR (phosphorylated mTOR), S6K1, pS6K1, 4E-BP1, p4E-BP1, AKT and pAKT showed that substitution of 25% of Met with Met-Met resulted in higher levels of p4E-BP1 and pS6K1 (Figure 8A,B). The ratios of pmTOR/mTOR, p4E-BP1/4E-BP1, pS6K1/S6K1, and pAKT/AKT were greater in response to substitution with 25% Met-Met (Figure 8C), which suggested the activated AKT/mTOR signaling pathway.

## 4. Discussion

We first developed a Met-deficient pregnant mouse model in which Met or Met-Met was supplemented by i.p. injection. The reason we used i.p. injection rather than gavage was that Met-Met is not stable in the digestive tract, as shown in stability tests in SGF and SIF. On the other hand, our results show that Met-Met is relatively stable in the serum for a longer period of time. In addition, our in vivo imaging assays show that the i.p.-injected Met-Met conjugated with fluorescein isothiocyanate, an amine-reactive derivative of fluorescein dye extensively used to label peptides [21], can be quickly absorbed and become invisible within 24 h, although most Free-FITC appears to accumulate in the injection site at 24 h. These observations support our method to supplement Met-Met by i.p. injection.

To study the role of Met-Met in reproductive performance, the best dose of free Met supplementation by i.p. injection was analyzed first. Met-deficient pregnant mice were i.p. injected daily with 15–45% of the Met in the control diet in experiment I. These doses were chosen based on research indicating that less than half of dietary Met could be absorbed into the blood stream after the digestion and absorption process [22]. Although all Met-deficient groups showed impaired reproductive performance, the i.p. supplementation of 35% Met had the least impact, retaining approximately 80% of the litter number and 67% of the average litter weight at day 17 of gestation compared with the control group. Excess Met supplementation in the maternal (55% and 65% Met) was also detrimental to embryo development, which may have been caused by an imbalance of AA and possible changes in endocrine parameters [23].

Then, 5–45% of the free Met in the 35% Met group was replaced with Met-Met. The substitution of 15–35% of Met with Met-Met improved the body weight of the dam at day 17 of gestation, with the 25% substitution being the top performer. The 25% substitution also resulted in higher birth weights of the litters born alive. These observations suggest that at certain levels, a large amount of Met-Met can be used not only by the body but also more efficiently than free Met.

To address how Met-Met may be used more efficiently than free Met in terms of reproductive performance, the FAAs concentrations in the maternal and fetal blood of the control, 35% Met, and 25% Met-Met groups were measured in experiment III. Dietary Met restriction resulted in 42% and 19% lower EAAs and 48% and 18% lower NEAAs in the maternal blood of the 35% Met and 25% Met-Met groups, respectively. In general, the concentrations of NEAAs were affected more disparate in the 35% Met group. As a consequence, the ratio of EAAs/NEAAs was increased in the 35% Met group compared with in the 25% Met-Met group. In addition, the lower concentrations of EAAs and NEAAs in the fetal blood of the 35% Met and 25% Met-Met groups may have led to slower embryo growth, as AAs are crucial for neonatal growth [24]. The improved fetal development of the 25% Met-Met group compared with the 35% Met group also supported the notion that the impaired embryo development in these two groups was mainly due to AAs deficiency, which may result from the lower cumulative food intake in these two groups compared with the control group. The concentrations of EAAs, NEAAs, and the majority of FAAs in the maternal and fetal blood were much higher in the 25% Met-Met group than in the 35% Met group and showed no differences from those in the control group.

Both the 35% Met and 25% Met-Met groups had lower maternal and fetal blood concentrations of Met, indicating that Met supplementation in these two groups could not match the Met supply of the control group. However, although the concentration of Met in the maternal blood was 68% lower in the 25% Met-Met group than in the 35% Met group, resulting from the injection of a lower amount of free Met, the Met concentration in the fetal blood was much higher in the 25% Met-Met group than in the 35% Met group. This difference suggests that at least some Met-Met is more efficiently absorbed as a whole by peptide transporters, such as PhT1 and PepT1, as we measured much higher expression levels of PhT1 and PepT1 in the 25% Met-Met group.

In general, the concentration of FAAs was substantially enriched in the fetal plasma compared to that in the maternal plasma in the three groups. Specifically, the embryo/maternal ratio was 15.3 and 11.8 for Gly in the 25% Met-Met and 35% Met groups, respectively. In the present study, the abundance of AA transporters in the placenta was consistent with the AA concentrations of the fetal blood of the three groups. Many of the AA transporters were analyzed, including system A (SLC38A1, SLC38A2 and SLC38A4), which accounts for most Na^+^-dependent neutral amino acid uptake by mammalian cells [25], and system L (SLC7A8), which mainly transports large branched and aromatic neutral amino acids [26], showed lower mRNA abundance levels in the 35% Met group and higher abundance levels in the 25% Met-Met group.

Another reason for impaired embryo development in the Met-deficient groups may have been the lower concentrations of insulin, glucose, and leptin in the maternal blood of these dams. The normal metabolism of carbohydrates, lipids, and proteins is critical for pregnancy. Insulin is the central endocrine hormone in the regulation of metabolism. A high physiological level of insulin is required to promote fetal growth and development, whereas low insulin levels, such as in diabetic pregnancy, may lead to an increased risk of congenital abnormalities [27]. The amount of glucose production in dams, which is regulated by insulin, is a predictor of newborn outcomes [28]. In the present study, the abundance levels of SLC2A1 (GLUT1), SLC2A4 (GLUT4) and SLC2A3 (GLUT3) in the placenta were higher in the 25% Met-Met group than those in the 35% Met group, suggesting a higher glucose supply for fetal development in the 25% Met-Met group. In addition, the maternal leptin levels were positively correlated with fetal and neonatal size [29] and consistent with the food intake in those animals. In the present study, Ala, the most abundant amino acid in plasma and an important gluconeogenic precursor in the liver [30], as well as other glycemic and insulinemic AAs (Val + Leu + Ile + Thr + Lys + Arg), showed a sharp decline in the 35% Met dams. These decreases in AAs might have contributed to the lower insulin and glucose levels in the 35% Met group. Consistently, the maternal plasma levels of insulin, glucose and leptin, as well as the insulinemic and glycemic AAs, were higher in the 25% Met-Met group than in the 35% Met group, supporting higher embryo development.

The enhanced reproductive outcomes and nutrient availability in the 25% Met-Met group may have been due to signaling molecule activation in the placenta. A key pathway in the regulation of placental growth and nutrient transport is the PI3K/AKT/mTOR signaling pathway, which is the master regulator of cell growth and protein synthesis, among other functions [31,32]. Changes in maternal nutrient availability, particularly AAs, seem to be the major mediators of mTOR [33], which in turn regulates placental amino acid transporters in human trophoblast cells and influences the trafficking of specific AAs across the plasma membrane [34,35]. Previous studies have shown that placental AA transport changes in the same direction as mTOR signaling in clinical conditions and animal models of altered fetal growth [31,36]. In this study, the expression and phosphorylation levels of mTOR signaling factors, including S6K1 and 4E-BP1, were reduced in the 35% Met group but increased in the 25% Met-Met group. We speculate that in our study, Met-Met or increased AA availability in the 25% Met-Met group may have activated the mTOR signaling pathway, which in turn regulated the expression of AAs and other nutrient transporters and stimulated nutrient transport into the fetal circulation.

## 5. Conclusions

In summary, this study reveals that Met-Met can be used more efficiently than free Met in mice to support fetal growth under Met deficiency. The major reason for the enhanced embryo development was probably due to the higher cumulative food intake in the 25% Met-Met group than the 35% Met group, resulting in higher availability of nutrients and the hormonal/signaling alterations. Met-Met changes the maternal AA profile, increasing the concentration of gluconeogenic AAs and insulin secretion from the dam. Met-Met enters the placental barrier through peptide transporters and is hydrolyzed to Met. Enhanced insulin signaling and Met in the cytosol activate the AKT/mTOR signaling pathway, which increases the expression of nutrient transporters in the placenta and thus nutrient availability in fetal blood to promote fetal growth and development. The current results highlight a new investigation field in human and other mammals to determine whether reproductive outcomes can be optimized by supplementing mothers with small peptides.

## Supplementary Materials

The following are available online at http://www.mdpi.com/2072-6643/10/9/1190/s1, Figure S1: Schematic diagram of the animal study design of Experiment I (A), Experiment II (B), and Experiment III (C), Table S1: Ingredients of the experimental diets, Table S2: Primers used in mRNA abundance analysis (A), and antibodies for western blot analysis (B), Table S3: Body weight (BW) (A), food intake (B), and cumulative food intake (C) in mice at day (E) 0 to 17 of gestation, Table S4: Methionine (Met) metabolites (nnmol/g) in the liver of mice with different free Met supplementation at day 17 of gestation, Table S5: Body weight (BW) (A), food intake (B), and cumulative food intake (C) during gestation period when different levels of methionine (Met) were supplemented in the form of methionyl-methionine (Met-Met), TableS6: Plasma concentrations of free amino acids (FAAs, μg/mL) in maternal and fetal on day 17 of gestation in mice with different sources of methionine (Met) supplementation (*n* = 8), Table S7: The mRNA abundance of nutrient transporters in placenta of mice with different sources of methionine (Met) supplementation on day 17 of gestation.
