# Supplementary material for: Methionine Partially Replaced by Methionyl-Methionine Dipeptide Improves Reproductive Performance over Methionine Alone in Methionine-Deficient Mice
Source: Nutrients. 2018 Sep 1;10(9):1190. doi: 10.3390/nu10091190 (PMC6165284; doi:10.3390/nu10091190)
Supplement: Supplementary file 1 [file nutrients-10-01190-s001.pdf]

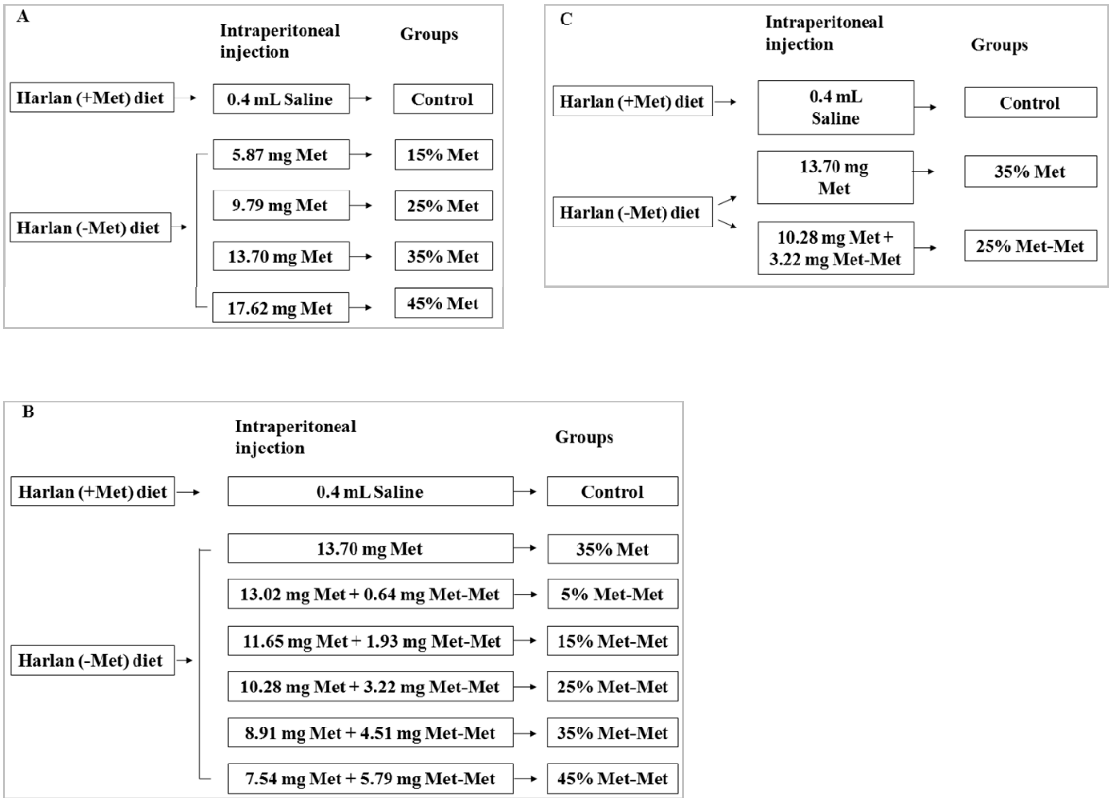

Supplemental Figure 1. Schematic diagram of the animal study design of Experiment I (**A**), Experiment II (**B**), and Experiment III (**C**). In total, 144 pregnant mice were included in in this study, with 8 mice per group in experiment I and II, 16 mice in experiment III. In experiment I, the control group (Control) was fed a methionine (Met) added diet based on Harlan [Harlan (+Met) diet], with daily intraperitoneal (i.p.) injection of 0.4 mL of saline, whereas the other four groups (15%, 25%, 35%, and 45% Met groups) were fed with the same diet, but without Met [Harlan (-Met) diet], with daily i.p. injection of 15, 25, 35 or 45% Met in the control diet (39.20 mg based on 4.8 g/d dry matter intake) in 0.4 mL of saline; In experiment II, groups 1 (Control) and 2 (35% Met) were treated as described for the control group and 35% Met group in experiment I, respectively. The other 5 groups (5, 15, 25, 35, and 45% Met-Met groups) were treated as described for the 35% Met group, except that 5, 15, 25, 35, or 45% free Met in the injected solution were replaced with Methionyl-methionine (Met-Met); In experiment III, mice were randomly divided into three groups and received treatments as described for the control, 35% Met, and 25% Met-Met groups in experiment II. At E 17, half of dams in the 35% Met and 25% Met-Met groups were switched to the control diet without i.p. injection and gave birth. Of all 144 mice included in the experiments, 18 mice were aborted and removed from the experiments.

Supplemental Table 1. Ingredients of the experimental diets.

Composition (g/kg)	Control	Treatment
L-alanine	3.5	3.5
L-arginine HCl	12.1	12.1
L-asparagine	6	6
L-aspartic acid	3.5	3.5
L-cystine	3.5	3.5
L-glutamic acid	35	35
Glycine	23.3	23.3
L-histidine HCl, monohydrate	4.5	4.5
L-isoleucine	8.2	8.2
L-leucine	11.1	11.1
L-lysine HCl	18	18
L-methionine	8.2	-
L-phenylalanine	11.6	11.6
L-proline	3.5	3.5
L-serine	3.5	3.5
L-threonine	8.2	8.2
L-tryptophan	1.8	1.8
L-tyrosine	3.5	3.5
L-valine	8.2	8.2
Sucrose	357.03	357.03
Corn starch	150	150
Maltodextrin	150	150
Soybean oil	80	80
Cellulose	30	30
Mineral Mix, AIN-93M-MX	35	35
Calcium phosphate, monobasic, monohydrate	8.2	8.2
Vitamin Mix, AIN-93-VX 2	10	10
Choline bitartrate	2.5	2.5
Vitamin K, menadione sodium bisulfite	0.05	0.05
Red food color	-	0.1
tert-Butylhydroquinone (TBHQ) antioxidant	0.02	0.02

Diet formulation is based on TD.99366 (a standard amino acid defined diet; Harlan). Vitamin, mineral and choline contents are based on recommendations for AIN-93G (Reeves PG. J Nutr 1997; 127:838S-41S); amino acid content is based on Rogers QR and Harper AE. (J. Nutr. 1965; 87:267-73).

Supplemental Table 2. Primers used in mRNA abundance analysis（A）, and antibodies for western blot analysis (B).

A

Gene	Primers((5'-3')	bp	Gene No.
	Amino acid transporters		
SLC38A1-F	AGCAACGACTCTAATGACTTCAC	81	NM_001166458.1
SLC38A-1R	CCTCCTACTCTCCCGATCTGA		
SLC38A2-F	GCTTGGCTTTATGGAGTCAAAGA	131	NM_175121.4
SLC38A2-R	TACCTGGATGCTACACAGAGG		
SLC38A4-F	GCGGGGACAGTATTCAGGAC	102	NM_027052.3
SLC38A4-R	GGAACTTCTGACTTTCGGCAT		
SLC1A1-F	CTTCCTACGGAATCACTGGCT	176	NM_009199.2
SLC1A-1R	CGATCAGCGGCAAAATGACC		
SLC7A1-F	CTTGGGCGCTGGTGTCTATG	173	NM_001301424.1
SLC7A1-R	CGTAGCTGTAGAGGTAGGCTG		
SLC1A4-F	GTGGCATCGCTGTTGCTTAC	237	NM_018861.3
SLC1A4-R	TTGCAGACGTAGTGAATGCGG		
SLC1A5-F	CATCAACGACTCTGTTGTAGACC	184	NM_009201.2
SLC1A5-R	CGCTGGATACAGGATTGCGG		
SLC7A9-F	GAGGAGACGGAGAGAGGATGA	172	NM_001199015.1
SLC7A9-R	CCCCACGGATTCTGTGTTG		
SLC7A5-F	ATATCACGCTGCTCAACGGTG	221	NM_011404.3
SLC7A5-R	CTCCAGCATGTAGGCGTAGTC		
SLC7A8-F	TTGCCCTGTCCACGTTTGG	158	NM_016972.2
SLC7A8-R	GGAGAGGCATGTGAAGAGCAG		
SLC43A2-F	TGCACCGCTGTGTTGGAAA	133	NM_001199284.1
SLC43A2-R	CCGTGCTGTTAGTGACATTCTC		
SLC7A7-F	AGCACCAAGTATGAAGTGGCT	124	NM_001253680.1
SLC7A7-R	ACACGCCATTAAGCAGGGAG		
SLC7A6-F	GCCTGCGTATGTCTGCTGA	117	NM_001357381.1
SLC7A6-R	GCCCATGATAATGATGGCAATGA		
SLC16A10-F	AGGTGCTCTTCATGTGCATTG	100	NM_028247.4
SLC16A10-R	TGGAGGTAGACCTTCTTCACAC		
	Glucose transporters		
SLC2A1-F	CAGTTCGGCTATAACACTGGTG	156	NM_011400.3
SLC2A1-R	GCCCCCGACAGAGAAGATG		
SLC2A3-F	ATGGGGACAACGAAGGTGAC	103	NM_011401.4
SLC2A3-R	GTCTCAGGTGCATTGATGACTC		
SLC2A4-F	GTGACTGGAACACTGGTCCTA	127	NM_001359114.1
SLC2A4-R	CCAGCCACGTTGCATTGTAG		
SLC2A5-F	CCAATATGGGTACAACGTAGCTG	116	NM_019741.3
SLC2A5-R	GCGTCAAGGTGAAGGACTCAATA		
SLC2A6-F	AACCGAGGGACTCGACTATGA	157	NM_001177627.1
SLC2A6-R	CAAGGCATACCCAAAGCTGAA		
SLC2A8-F	CCCTTCGTGACTGGCTTTG	138	NM_019488.5
SLC2A8-R	TGGGTAGGCGATTTCCGAGAT		
SLC2A9-F	GTACCCCTATCTGATGGAGGC	127	NM_001102415.1
SLC2A9-R	TGCACACACATACTCTGAAAAGC		
SLC2A10-F	GCCTGACCTTCGGATATGAGC	165	NM_130451.3
SLC2A10-R	TGCCATAGCAGTCAATGAGGA		
SLC2A12-F	GTACCTGTTGAAAACACAGAGGG	136	NM_178934.4
SLC2A12-R	TCAGGAACGTAAACATGCTGG		
SLC5A11-F	ATGCGGCTGACATCTCAGTC	247	NM_146198.2
SLC5A11-R	ACCAAGGCGTTCCATTCAAAG		
	Reference gene		
β-actin-F	GGCTGTATTCCCCTCCATCG	154	NM_007393.5
β-actin-R	CCAGTTGGTAACAATGCCATGT		

B

Protein	Supplier	Item NO.
PHT1	Abnova (Taipei, Taiwan)	20900
PepT1	Abcam (Cambridge, MA, USA)	78020
PepT2	Abcam (Cambridge, MA, USA)	83771
p-mTOR (Ser 2448)	Cell Signaling Technology (Danvers, MA, USA)	5536
mTOR	Cell Signaling Technology (Danvers, MA, USA)	2983
p-4E-BP1 (Thr 37/46)	Cell Signaling Technology (Danvers, MA, USA)	2855
4E-BP1	Cell Signaling Technology (Danvers, MA, USA)	4923
p-S6K1 (Thr 389)	Cell Signaling Technology (Danvers, MA, USA)	9234s
S6K1	Cell Signaling Technology (Danvers, MA, USA)	9202,
p-AKT (Ser 473)	Cell Signaling Technology (Danvers, MA, USA)	4060
AKT	Cell Signaling Technology (Danvers, MA, USA)	4691
β-actin	Abcam (Cambridge, MA, USA)	8226

Supplemental Table 3. Body weight (BW) (A), food intake (B), and cumulative food intake (C) in mice at day (E) 0 to 17 of gestation. The control group of mice (Control) was fed a diet with free methionine (Met), whereas the 5 - 45% Met groups were fed a Met-deficient diet but supplemented with 5, 15, 25, 35, and 45% Met in the control diet by intraperitoneal injection from conception.

A

BW (g)	E 0	E 2	E 5	E 8	E 11	E 14
Control (n = 7)	29.85 ± 1.25 ^{d}	29.74 ± 1.14 ^{d}	31.46 ± 1.02 ^{d}	34.28 ± 1.12 ^{c}	37.43 ± 1.29 ^{b}	44.56 ± 0.96 ^{a}
15% Met (n = 6)	30.29 ± 1.17	30.95 ± 0.72	30.25 ± 0.89	29.31 ± 0.68 ***	29.27 ± 0.75 ***	31.43 ± 0.64 ***
25% Met (n = 7)	30.98 ± 0.97	30.43 ± 0.68	29.38 ± 1.09	28.94 ± 1.06 ***	29.24 ± 1.44 ***	32.03 ± 1.28 ***
35% Met (n = 7)	30.02 ± 0.91 ^{b}	29.74 ± 0.99 ^{b}	29.52 ± 0.89 ^{b}	29.63 ± 0.78 $^{***' b}$	31.62 ± 0.84 ^{***,b}	35.90 ± 0.91 ^{***,a}
45% Met (n = 6)	30.33 ± 1.18 $^{a, b}$	29.17 ± 1.07 ^{b}	28.87 ± 0.86 ^{b}	29.83 ± 1.06 ^{***,b}	29.56 ± 0.99 ^{***,b}	32.88 ± 1.06 ^{***,a}

B

Food intake (g)	E 2	E 5	E 8	E 11	E 14
Control (n = 7)	3.87 ± 0.22 ^{c}	3.51 ± 0.17 ^{c}	3.57 ± 0.17 ^{c}	5.48 ± 0.20 ^{b}	6.15 ± 0.21^{a}
15% Met (n = 6)	3.22 ± 0.32 ^{b}	3.09 ± 0.37 ^{b}	2.97 ± 0.26 ^{b}	3.75 ± 0.43 $^{**, a, b}$	4.23 ± 0.26 $^{***, a}$
25% Met (n = 7)	3.38 ± 0.31 ^{b}	3.30 ± 0.23 ^{b}	3.44 ± 0.24 ^{b}	4.20 ± 0.25 $^{**, a}$	4.37 ± 0.18$^{***, a}$
35% Met (n = 7)	3.15 ± 0.26 ^{b}	3.44 ± 0.30 ^{b}	3.23 ± 0.22 ^{b}	4.33 ± 0.30$^{**, a}$	4.90 ± 0.26$^{***, a}$
45% Met (n = 6)	3.30 ± 0.28 ^{d}	3.11 ± 0..26 $^{d`}$	3.52 ± 0.18 $^{b, c, d}$	4.14 ± 0.18$^{**, a, b}$	4.72 ± 0.19$^{***, a}$

C

Cumulative food intake (g)	E 5	E 8	E 11	E 14
Control (n = 7)	7.38 ± 0.37 ^{d}	10.94 ± 0.37^{c}	16.4 ± 0.48 ^{b}	22.6 ± 0.50 ^{a}
15% Met (n = 6)	6.31 ± 0.51 ^{d}	9.29 ± 0.47$^{*, c}$	13.0 ± 0.65$^{***, b}$	17.1 ± 0.51 $^{***, a}$
25% Met (n = 7)	6.68 ± 0.45 ^{d}	10.1 ± 0.61^{c}	14.3 ± 0.73$^{*, b}$	18.7 ± 0.75 $^{***, a}$
35% Met (n = 7)	6.60 ± 0.48 ^{d}	9.83 ± 0.65^{c}	14.2 ± 0.71$^{*, b}$	19.1 ± 0.73 $^{***, a}$
45% Met (n = 6)	6.41 ± 0.44 ^{d}	9.94 ± 0.49^{c}	14.1 ± 0.41$^{*, b}$	18.8 ± 0.54 $^{***, a}$

Data are expressed as the mean ± SEM. *, **, *** significantly different from the control group at the same stage of gestation at p <.05 or p<.01 or p<.001, respectively. Values with different letters differ significantly (p <.05) between different stages of gestation within group.

Supplemental Table 4. Methionine (Met) metabolites (nnmol/g) in the liver of mice with different free Met supplementation at day 17 of gestation. The control group of mice (Control) were fed a diet with Met, and the 15 - 45% Met groups were fed a Met-restricted diet supplemented with 15, 25, 35, and 45% Met in the control diet by intraperitoneal injection.

	Met	SAM	SAH	Cysteine	Homocysteine
Control (*n* = 7)	64.53±1.42	78.67±0.86	46.78±1.70	81.66±2.27	5.74±0.13
15% Met (*n* = 6)	63.61±1.30	81.07±0.72	43.34±0.78	85.34±1.21	5.62±0.11
25% Met (*n* = 7)	67.67±1.38	77.77±1.08	46.82±1.30	80.58±1.66	5.69±0.10
35% Met (*n* = 7)	64.99±1.24	79.56±1.23	49.40±1.06	83.85±1.97	5.96±0.13
45% Met (*n* = 6)	68.14± 2.20	77.54±1.26	49.88±1.20	81.37±2.12	6.03±0.15

SAM, adenosylmethionine; SAH, adenosylhomocysteine.

Supplemental Table 5. Body weight (BW) (A), food intake (B), and cumulative food intake (C) during gestation period when different levels of methionine (Met) were supplemented in the form of methionyl-methionine (Met-Met). The control group of mice (Control) was fed a diet with Met, and the 35% Met group was fed a Met-deficient diet but supplemented with 35% of Met in the control diet by intraperitoneal injection. In 5, 15, 25, 35, and 45% Met-Met groups, 5, 15, 25, 35, and 45% Met in 35% Met group was replaced with Met-Met.

A

BW (g)	E 0	E 2	E 5	E 8	E 11	E 14
Control (*n* = 8)	30.27±1.10^{d}	30.14±0.74^{d}	32.24±0.82$^{c\ d}$	34.67±0.84^{c}	37.85±0.68^{b}	45.05±0.98^{a}
35% Met (*n* = 6)	30.44±1.13^{b}	30.21±1.52^{b}	30.02±1.37^{b}	29.64±1.39^{**,b}	31.56±0.96^{***,ab}	34.31±0.99^{***,a}
5% Met-Met (*n* = 6)	28.54±0.91^{b}	28.16±0.85^{b}	29.12±1.13^{b}	29.56±1.34^{**,b}	31.33±1.40^{***,b}	34.86±1.24^{***,a}
15% Met-Met (*n* = 7)	29.75±1.05^{b}	29.99±1.06^{b}	30.54±1.20^{b}	31.36±1.22^{*,b}	33.14±1.23^{**,b}	37.04±1.27^{***,a}
25% Met-Met (*n* = 7)	29.91±0.88^{c}	29.81±0.85^{c}	30.89±0.99^{c}	31.36±1.22^{c}	34.41±1.04^{*,b}	38.54±1.44^{***,a}
35% Met-Met (*n* = 7)	30.79±0.60^{b}	30.61±0.73^{b}	30.71±1.02^{b}	31.34±1.05^{*,b}	32.93±1.35^{**,b}	36.00±1.29^{***,a}
45% Met-Met (*n* = 6)	30.06±1.11^{b}	29.78± 0.83^{b}	29.92±0.73^{b}	30.69±0.94^{*,b}	32.08±0.89^{**,ab}	34.29±0.87^{***,a}

B

Food intake (g)	E 2	E 5	E 8	E 11	E 14
Control (*n* = 8)	3.45±0.24^{c}	3.67±0.23^{c}	3.46±0.18^{c}	5.61±0.14^{b}	6.20±0.16^{a}
35% Met (*n* = 6)	3.24±0.27^{b}	3.22±0.23^{b}	3.30±0.32^{b}	4.04±0.25^{***,a}	4.40±0.22^{***,a}
5% Met-Met (*n* = 6)	3.11±0.23^{bc}	3.38±0.22^{b}	3.53±0.22^{b}	4.14±0.42^{***,ab}	4.60±0.17^{***,a}
15% Met-Met (*n* = 7)	3.25±0.17^{b}	3.39±0.21^{b}	3.58±0.20^{b}	4.55±0.21^{***,a}	4.46±0.21^{***,a}
25% Met-Met (*n* = 7)	3.56±0.22^{b}	3.40±0.21^{b}	3.47±0.14^{b}	4.47±0.15^{***,a}	4.69±0.20^{***,a}
35% Met-Met (*n* = 7)	3.49±0.17^{cd}	3.37±0.25^{cd}	3.71±0.20^{bc}	4.05±0.16^{***,b}	4.60±0.16^{***,a}
45% Met-Met (*n* = 6)	3.50±0.22^{b}	3.73±0.21^{b}	3.70±0.17^{b}	3.96±0.19^{***,ab}	4.48±0.28^{***,a}

C

Cumulative food intake (g)	E 5	E 8	E 11	E 14
Control (*n* = 8)	7.11±0.33^{d}	10.6±0.37^{c}	16.2±0.38^{b}	22.4±0.27^{a}
35% Met (*n* = 6)	6.46±0.42^{d}	9.75±0.22^{c}	13.8±0.32^{***,b}	18.2±0.36^{***,a}
5% Met-Met (*n* = 6)	6.50±0.30^{d}	10.0±0.28^{c}	14.2±0.44^{***,b}	18.8±0.43^{***,a}
15% Met-Met (*n* = 7)	6.95±0.22^{d}	10.5±0.34^{c}	15.1±0.45^{*,b}	19.5±0.54^{***,a}
25% Met-Met (*n* = 7)	6.96±0.36^{d}	10.4±0.38^{c}	14.9±0.37^{*,b}	19.6±0.42^{***,a}
35% Met-Met (*n* = 7)	6.86±0.35^{d}	10.6±0.35^{c}	14.7±0.34^{**,b}	19.2±0.27^{***,a}
45% Met-Met (*n* = 6)	7.23±0.34^{d}	10.9±0.36^{c}	14.9±0.39^{*,b}	19.4±0.45^{***,a}

Data are expressed as the mean ± SEM, *n* = 6–8. *, **, *** significantly different from the control group at the same stage of gestation at $p < 0.05$ or $p < 0.01$ or $p < 0.001$, respectively. Values with different letters differ significantly ($p < 0.05$) between different stages of gestation within group.

Supplemental Table 6. Plasma concentrations of free amino acids (FAAs, µg/ml) in maternal and fetal on day 17 of gestation in mice with different sources of methionine (Met) supplementation (n = 8). In Control group, pregnant mice were fed a diet containing free Met. In the 35% Met group, pregnant mice were fed a Met-deficient diet with intraperitoneal injection of 35% Met contained in the control diet. The 25% methionyl-methionine (Met-Met) group was treated as 35% Met group, except that 25% of injected Met was replaced with Met-Met.

FAAs (µg/ml)	Dams			Embryos		
	Control	35% Met	25% Met-Met	Control	35% Met	25% Met-Met
EAAs	227 ± 2.50 [a]	132 ± 7.45 [c]	184 ± 3.57 [b]	326 ± 5.70 [a]	21.3 ± 11.0 [c]	297 ± 11.20 [b]
Arg	6.53 ± 0.39 [a]	4.85 ± 0.20 [b]	5.33 ± 0.45 [b]	10.6 ± 0.69 [a]	5.43 ± 0.32 [b]	11.5 ± 0.44 [a]
His	8.79 ± 0.17 [a]	5.72 ± 0.78 [b]	7.75 ± 0.20 [a]	15.3 ± 1.00	12.5 ± 0.81	11.8 ± 1.59
Ile	9.07 ± 0.28 [a]	3.62 ± 0.25 [c]	7.04 ± 0.54 [b]	14.6 ± 0.96 [a]	7.92 ± 0.72 [b]	13.3 ± 1.29 [a]
Leu	19.4 ± 0.68 [a]	5.71 ± 0.69 [c]	17.1 ± 0.64 [b]	34.4 ± 1.94 [a]	20.3 ± 1.24 [b]	33.4 ± 2.95 [b]
Lys	88.4 ± 1.45 [a]	51.7 ± 3.18 [b]	87.4 ± 1.85 [a]	134 ± 2.86 [a]	78.9 ± 4.55 [c]	117 ± 1.55 [b]
Met	26.5 ± 1.61 [a]	21.5 ± 1.50 [b]	6.92 ± 0.36 [c]	15.1 ± 0.40 [a]	4.66 ± 0.14 [c]	11.8 ± 0.55 [b]
Phe	6.96 ± 0.25 [a]	4.62 ± 0.55 [b]	4.64 ± 0.12 [b]	23.5 ± 0.95	22.3 ± 1.57	10.6 ± 1.82
Thr	30.9 ± 0.72 [a]	22.9 ± 3.56 [b]	24.2 ± 1.12 [b]	43.9 ± 1.87 [a]	34.9 ± 2.38 [b]	42.3 ± 2.49 [a]
Val	30.6 ± 0.73 [a]	11.6 ± 0.90 [c]	24.1 ± 1.34 [b]	34.5 ± 1.35 [a]	24.5 ± 1.93 [b]	35.7 ± 2.62 [a]
NEAAs	286 ± 4.31 [a]	149 ± 10.32 [c]	233 ± 5.45 [b]	409 ± 13.3 [a]	282 ± 8.57 [c]	369 ± 13.8 [b]
Ala	79.5 ± 2.78 [a]	37.0 ± 4.62 [c]	66.7 ± 2.92 [b]	105 ± 6.69 [a]	73.1 ± 4.28 [b]	103 ± 7.02 [a]
Asp	169 ± 4.41 [a]	90.7 ± 5.00 [c]	138 ± 3.36 [b]	185 ± 3.46 [a]	133 ± 5.29 [c]	164 ± 8.00 [b]
Cys	11.3 ± 0.40 [a]	9.95 ± 0.31 [b]	9.58 ± 0.18 [b]	17.1 ± 0.65 [a]	10.1 ± 0.55 [c]	12.9 ± 0.73 [b]
Glu	5.15 ± 0.22 [a]	1.69 ± 0.15 [c]	2.93 ± 0.16 [b]	8.30 ± 0.35 [a]	4.77 ± 0.29 [c]	5.98 ± 0.27 [b]
Gly	2.62 ± 0.12 [a]	2.26 ± 0.08 [b]	1.69 ± 0.08 [c]	24.0 ± 3.14	26.6 ± 1.58	25.2 ± 0.93
Ser	8.52 ± 0.20 [a]	4.97 ± 0.46 [c]	7.10 ± 0.26 [b]	46.2 ± 2.33 [a]	30.7 ± 2.66 [b]	42.9 ± 2.18 [a]
Tyr	10.3 ± 0.34 [a]	2.54 ± 0.44 [c]	7.20 ± 0.33 [b]	23.0 ± 0.79 [a]	13.2 ± 0.40 [c]	15.0 ± 0.52 [b]
Total FAA	513 ± 6.12 [a]	281 ± 17.1 [c]	418 ± 8.36 [b]	735 ± 17.7 [a]	493 ± 17.4 [c]	666 ± 22.8 [b]

EAAs, essential amino acids; NEAAs, nonessential amino acids; Data are expressed as the mean ± SEM. The different letters indicate significant differences, $p < 0.05$.

Supplemental Table 7. The mRNA abundance of nutrient transporters in placenta of mice with different sources of methionine (Met) supplementation on day 17 of gestation. In Control group, pregnant mice were fed a diet containing free Met. In the 35% Met group, pregnant mice were fed a Met deficient diet with intraperitoneal injection of 35% Met contained in the control diet. The 25% methionyl-methionine (Met-Met) group was treated as described for the 35% Met group, except that 25% of injected Met was replaced with Met-Met. Three placentas from each groups were selected randomly, $n = 3$. The experiment was repeated 3 times.

Gene	Description	Control	35% Met	25% Met-Met
AA transporters				
SLC38A1	Neutral amino acid transporter	1.00 ± 0.05 [a]	0.68 ± 0.11 [b]	0.82 ± 0.12 [a,b]
SLC38A2	Neutral amino acid transporter	1.00 ± 0.05 [a]	0.46 ± 0.11 [b]	0.99 ± 0.13 [a,b]
SLC38A4	Neutral amino acid transporter	1.00 ± 0.05 [a]	0.561 ± 0.13 [b]	0.931 ± 0.17 [a,b]
SLC1A1	Glutamate transporter	1.00 ± 0.04 [a]	0.59 ± 0.10 [b]	0.53 ± 0.05 [b]
SLC1A3	Glutamate transporter	1.00 ± 0.01 [a]	0.65 ± 0.07 [b]	0.58 ± 0.06 [b]
SLC7A1	L-arginine transporter	1.00 ± 0.03	1.03 ± 0.04	0.94 ± 0.10
SLC1A4	Neutral amino acid transporter	1.00 ± 0.01	0.90 ± 0.11	0.99 ± 0.15
SLC1A5	Neutral amino acid transporter	1.00 ± 0.01	1.09 ± 0.08	1.14 ± 0.06
SLC7A9	Cationic amino acid transporter	1.00 ± 00.03 [a]	0.54 ± 0.04 [b]	0.97 ± 0.25 [a,b]
SLC7A5	Branched-chain and aromatic amino acid transporter	1.00 ± 0.04	0.21 ± 0.16	1.18 ± 0.09
SLC7A8	Branched-chain and aromatic amino acid transporter	1.00 ± 0.04 [a]	0.10 ± 0.04 [c]	0.48 ± 0.11 [b]
SLC43A2	Leu, Phe, Val, Met transporter	1.00 ± 0.03 [a]	0.54 ± 0.08 [b]	0.88 ± 0.22 [a,b]
SLC43A1	Leu, Phe, Val, Met transporter	1.00 ± 0.01 [a]	0.58 ± 0.05 [b]	0.84 ± 0.11 [a]
SLC7A7	Cationic amino acid transporter	1.00 ± 0.02	0.75 ± 0.16	0.76 ± 0.11
SLC7A6	Cationic amino acid transporter	1.00 ± 0.03 [a]	0.12 ± 0.05 [c]	0.51 ± 0.11 [b]
SLC16A10	Monocarboxylic acid transporter	1.00 ± 0.03 [a]	0.64 ± 0.09 [b]	0.79 ± 0.11 [a,b]
SLC7A3	Cationic amino acid transporter	1.00 ± 0.01 [a]	0.59 ± 0.13 [b]	0.63 ± 0.14 [b]
SLC7A4	Cationic amino acid transporter	1.00 ± 0.02 [a]	0.38 ± 0.11 [b]	0.89 ± 0.17 [a]
Glucose transporters				
SLC2A1	Glucose, galactose, mannose transporter	1.00 ± 0.11[a]	0.58 ± 0.13[b]	0.77 ± 0.11 [a,b]
SLC2A3	Glucose, galactose, mannose, maltose transporter	1.00 ± 0.02[a]	0.49 ± 0.09[b]	0.71 ± 0.17 [a,b]
SLC2A4	Glucose/fructose transporter	1.00 ± 0.01[a]	0.59 ± 0.12[b]	0.73 ± 0.11 [a,b]
SLC2A5	Glucose transporter	1.00 ± 0.01 [a]	0.40 ± 0.04[c]	0.73 ± 0.10 [b]
SLC2A6	Glucose transporter	1.00 ± 0.01	0.88 ± 0.10	1.19 ± 0.28
SLC2A8	Glucose and fructose transporter	1.00 ± 0.02	0.83 ± 0.22	0.91 ± 0.26
SLC2A9	Glucose and galactose transporter	1.00 ± 0.05	0.88 ± 0.25	0.97 ± 0.32
SLC2A10	Glucose transporter	1.00 ± 0.02[a]	0.46 ± 0.11[b]	0.80 ± 0.16 [a]
SLC2A12	Glucose transporter	1.00 ± 0.04	0.79 ± 0.26	0.98 ± 0.26
SLC5A11	Glucose, galactose, mannose transporter	1.00 ± 005	0.73 ± 0.19	0.75 ± 0.22

Data are expressed as the mean ± SEM. The different letters indicate significant differences, $p < 0.05$.
